# Adult worm exclusion and histological data of dogs repeatedly infected with the cestode *Echinococcus multilocularis*

**DOI:** 10.1016/j.dib.2020.105353

**Published:** 2020-03-02

**Authors:** Hirokazu Kouguchi, Hidefumi Furuoka, Takao Irie, Jun Matsumoto, Ryo Nakao, Nariaki Nonaka, Yasuyuki Morishima, Kazuhiro Okubo, Kinpei Yagi

**Affiliations:** a Department of Infectious Diseases, Hokkaido Institute of Public Health, N19 W12, Kita-Ku, Sapporo, Hokkaido, 060-0819, Japan; b Department of Basic Veterinary Medicine, Obihiro University of Agriculture and Veterinary Medicine, Inada-cho, Obihiro, Hokkaido, 080-8555, Japan; c Laboratory of Medical Zoology, College of Bioresource Sciences, Nihon University, Fujisawa, Kanagawa 252-0880, Japan; d Laboratory of Parasitology, Department of Disease Control, Graduate School of Veterinary Medicine, Hokkaido University, Sapporo, Hokkaido, 001-0020, Japan; e Department of Parasitology, National Institute of Infectious Diseases, Tokyo, 162-8640, Japan

**Keywords:** *Echinococcus multilocularis*, Vaccine, Repeated infection, Dog, Final host, Mucosal immunity, Histological analysis

## Abstract

The data presented in this article are related to a previously published research article titled “The timing of worm exclusion in dogs repeatedly infected with the cestode *Echinococcus multilocularis*” (2016) [1]. This data describe a comparison of worm exclusion in the early stage of infection (1 day and 6 days post-infection) between dogs infected for the first time (control group) and dogs repeatedly infected with the parasite 4 times (repeated infection groups). We observed that 6 days post reinfection, the number of adult worms in repeated-infection groups decreased by 88.7% compared with the control group. Histological analysis comparison of the small intestinal mucosa from healthy, first infected, and repeatedly infected dogs are also reported. We observed no clear pathological abnormality, except the shortening of microvillus in reinfected dogs. However, eosinophil accumulation and eosinophilic ulcers were observed in some reinfected dogs. This data could be useful as preliminary data to develop a final host vaccine for this parasite.

**Table:** Specifications Table

Subject	Immunology and Microbiology
Specific subject area	Parasitology
Type of data	Table Figure
How data were acquired	The number of worms in dogs infected with *E. multilocularis* was counted by stereomicroscope. The histological analysis was performed by optical microscopic observation.
Data format	Raw Analyzed
Parameters for data collection	Worm numbers were compared between the first-infection group (dogs infected with *E. multilocularis* for the first time) and repeated-infection group (dogs repeatedly infected with the parasite 4 times). A comparison of the histological profile was performed between healthy, first infection, and repeated infection groups.
Description of data collection	Fourteen dogs were divided into two groups as followed: first-infection group (dogs infected with *E. multilocularis* for the first time; n = 4) and repeated-infection group (dogs repeatedly infected with this parasite 4 times; n = 10). Necropsy of these 10 dogs repeatedly infected with the parasite was performed at 1 day and 6days post reinfection. Sample tissue was taken out from the central part of the small intestine.
Data source location	Hokkaido Institute of Public Health, Sapporo, Japan, 43°04′58.804″N; 141°19′59.769″E″
Data accessibility	With the article
Related research article	Kouguchi H, Irie T, Matsumoto J, Nakao R, Sugano Y, Oku Y, Yagi K, The timing of worm exclusion in dogs repeatedly infected with the cestode Echinococcus multilocularis, J Helminthol, 10.1017/S0022149X15001169

**Table:** 

**Value of the Data** • The data of histopathology changes may facilitate understanding of worm exclusion in dogs infected with *Echinococcus.* • The data on worm exclusion during the early re-infection stage may contribute to the development of a vaccine for final hosts living at highly endemic areas where human echinococcosis is prevalent. • This data could contribute to clarify the exclusion mechanisms of various canine pathogens.

## 1 Data description

The data presented here were collected to elucidate the mechanisms underlying worm exclusion in dogs repeatedly infected with *E. multilocularis*. The decrease in the number of adult worms in repeatedly infected dogs is shown in Table 1. Dogs infected for the first time (control group) showed a worm burden ranging from 29,950 to 55,175. Six days post reinfection, the worm burden in dogs repeatedly infected four times with *E. multilocularis* (dogs R1-R5) showed a burden ranging from 0 (below detection limit) to 9450. Thus, the mean number of worms significantly decreased by 88.7% in this group, compared to the control (*p* < 0.05). One day post-infection, the worm burden of dogs repeatedly infected four times ranged from 2650 to 64,738 (dogs R6-R10). Compared to the control, the mean number of worms decreased by 41.1%, although this decrease was not statistically significant, probably due to the large distribution of worm numbers among the dogs in this group.

**Table 1:** Infection regimes to show the number of worms of *Echinococcus multilocularis*. ∗Statistical significance (p < 0.05).

Dog group	Times of infection	Worm number	Age of dogs (at final infection)	Period of infection (Days)	Days between infections
Control					
C1	1	43,175	8	6	
C2	1	41,075	8	6	
C3	1	29,950	8	6	
C4	1	55,175	8	6	
Average		42,344∗			
Reinfection					
R1	4	9450	8	30, 34, 35, 6	14, 8, 14
R2	4	N.D	8	30, 34, 35, 6	14, 8, 14
R3	4	11,450	8	30, 34, 35, 6	14, 8, 14
R4	4	3050	8	30, 34, 35, 6	14, 8, 14
R5	4	50	8	35, 35, 35, 6	7, 7, 14
Average		4800∗			
Reinfection					
R6	4	2738	8	35, 35, 35, 1	28, 7, 18
R7	4	2650	8	35, 35, 35, 1	7, 7, 18
R8	4	14,750	8	35, 35, 35, 1	9, 7, 20
R9	4	64,738	8	35, 35, 35, 1	9, 7, 20
R10	4	39,905	8	35, 35, 35, 1	7, 7, 14
Average		24,956			

In our previous report [1], through observations of mucus feces or diarrhea, we have proposed that adult worm exclusion occurs at an early stage in dogs repeatedly infected with *E. multilocularis*. The data presented here directly demonstrated that adult worm exclusion was almost complete by day 6 post–re-infection. Modulation of host responses is an important strategy whereby parasites ensure to settle and persist in the host [2]. Therefore, exclusion of adult worms at an early stage of infection may be crucial for the development of a final host vaccine.

**Fig. 1:**
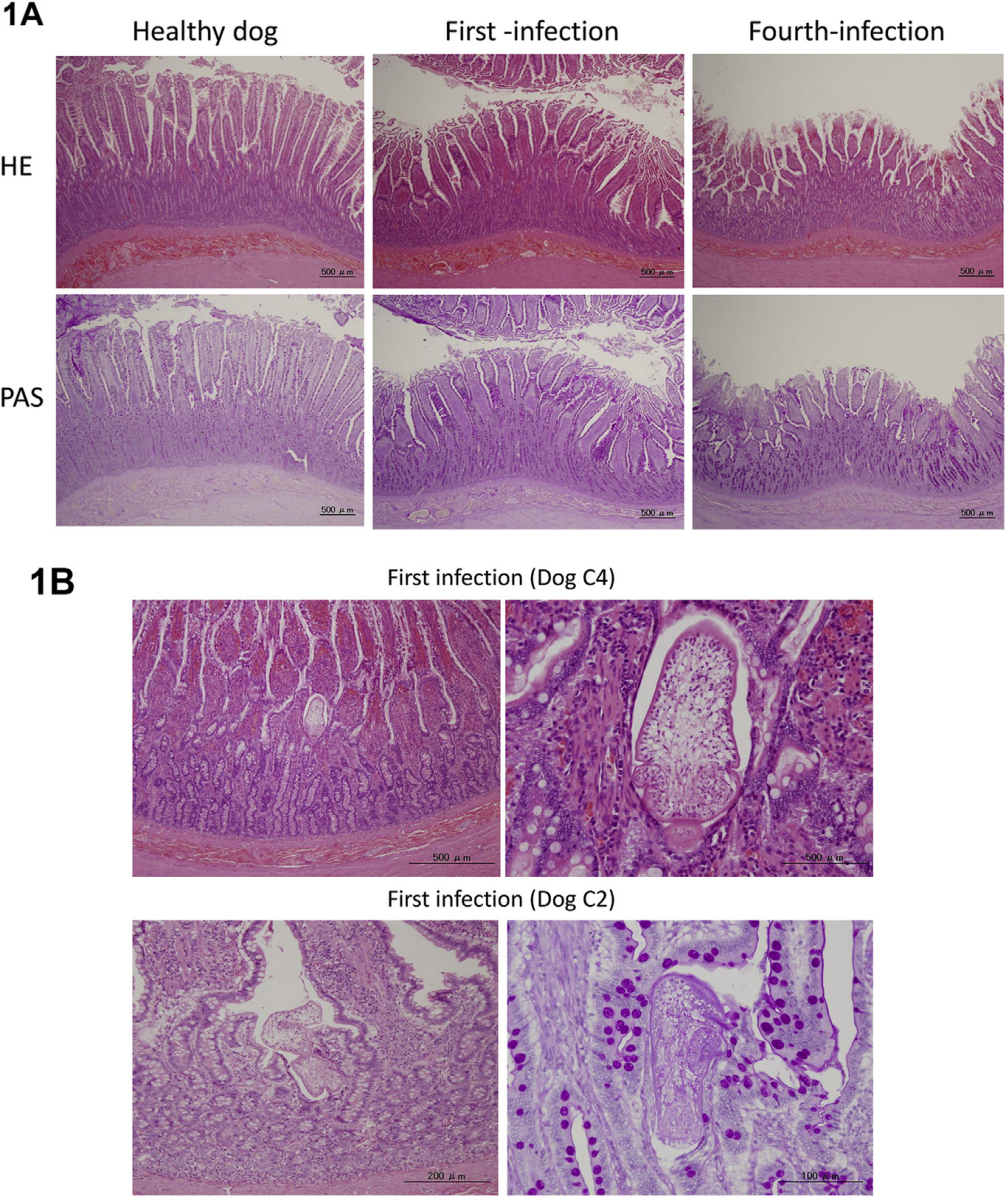

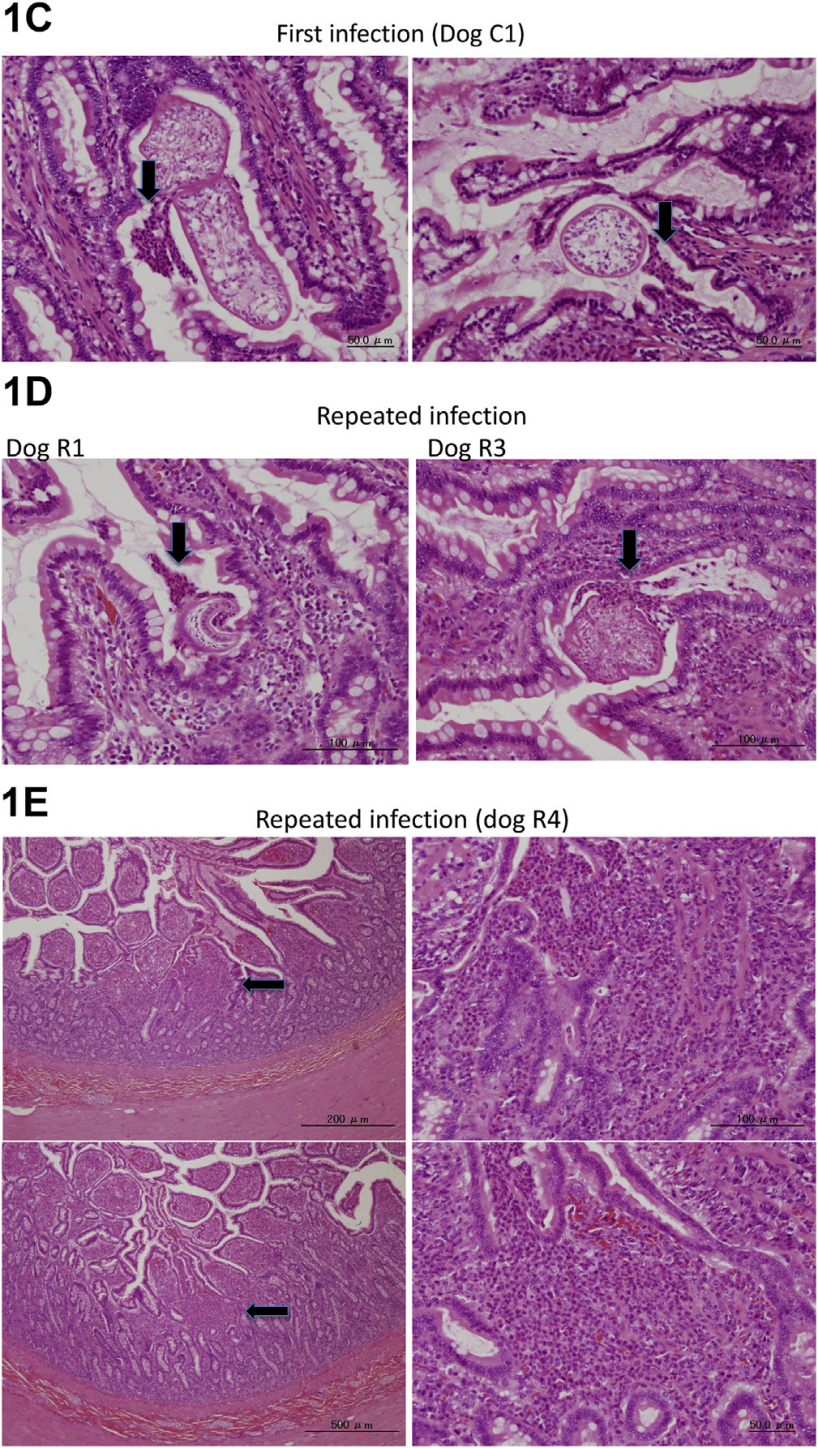
Histological analysis of *E. multiocularis*-infected dogs. Comparison of the small intestinal mucosa from healthy, first-infected, and fourth-infected dogs necropsied 6 days post-infection. Upper and lower panels show the HE and PAS staining, respectively (A). There are no clear responses in the small intestinal mucosa of first-infected dogs (B). In some dogs, eosinophil accumulation around infected worms was found, as shown in panels (C) and (D). Eosinophilic ulcer observed in dog R4 is shown in (E). The arrows indicate each eosinophilic response.

A typical histological image of the small intestinal mucosa of each group is shown in Fig. 1. Fig. 1A shows the comparison of a healthy dog, a dog infected with this parasite for the first time, and a dog repeatedly infected with the parasite four times (6 days post-infection). The microvillus of the infected dogs was shortened compared with the healthy dog. There was no clear histopathological abnormality in the first infected- and in the reinfected dogs. The histopathological findings in the small intestinal mucosa of dogs infected for the first time are shown in Fig. 1B and C. Fig. 1B shows no eosinophil accumulation around the worms in dog C2 and dog C4, while eosinophil infiltration was observed in some dogs (such as dog C1). Fig. 1D shows eosinophil accumulation around the worms in dogs R1 and R3, belonging to the repeatedly infected group observed 6 days post-infection. Fig. 1E shows an eosinophilic ulcer observed in dog R4.

## 2 Experimental design, materials, and methods

This study was performed in strict accordance with the National Institutes of Health guide for the care and use of laboratory animals, and the protocol for the experiments on the animals was approved by the ethics committee of the Hokkaido Institute of Public Health (permit number: K25–2 and K29-4). All surgeries were performed after sodium pentobarbital anesthesia, and every effort was made to minimize animal suffering.

Fourteen dogs were divided into 3 groups as followed: dogs infected with *E. multilocularis* for the first time (first infection group, n = 4), dogs repeatedly infected with the parasite 4 times and necropsied 6 days after the final infection (n = 5) and dogs repeatedly infected with the parasite 4 times and necropsied 1 day after the final infection (n = 5).

For the first-infection group, 100,000 protoscoleces were orally administered. In this group, the infection continued for 6 days. In the two groups of dogs infected repeatedly, 500,000 protoscoleces were administered orally in the first, second, and third infections. To terminate each infection, 100 mg of praziquantel (2 tablets of Droncit®, Bayer-Animal Health) was administered 30, 34, and/or 35 days post-infection (dpi), as summarized in Table 1. After deworming, dogs were re-infected by administering protoscoleces at intervals ranging from 7 to 28 days (Table 1). For the fourth and final infection, 100,000 protoscoleces were orally administered. Following the final infection, the dogs were euthanized and the small intestine was removed, divided into six sections, and incubated in 100 ml of Dulbecco's modified Eagle's medium (DMEM) at 4 °C for 7 days. Naturally released and scraped worms were counted with a dilution of 1:125.

## 3 Materials

*E. multilocularis* (Nemuro strain) was obtained from a dog-cotton rat life cycle routinely maintained at the Hokkaido Institute of Public Health (Sapporo, Japan). Protoscoleces were collected from cysts developed in cotton rats 5–14 months after the infection. Beagle dogs and small intestine tissues from healthy dogs were purchased from KITAYAMA LABES CO., LTD. (Ina, Nagano, Japan).

### 3.1 Histological analysis

All small intestine tissue used in this study were fixed in 10% formalin-PBS and embedded in paraffin wax. Cryosections were cut on a Retoratome REM-710 microtome (Yamato Kohki Co., Ltd., Japan) at −25 °C with a thickness of 4 μm and mounted on slides. The samples were further stained by HE and/or PAS staining and then washed with distilled water.

### 3.2 Data analysis

Data analysis was performed using the R software, version 2.15.2 (R Foundation for Statistical Computing, Vienna, Austria). We performed a Shapiro–Wilk normality test and observed that the data were not normally distributed. Therefore, the non-parametric Mann–Whitney *U* test was used to compare the number of parasites between the groups. A *p-value* < 0.05 was considered statistically significant.
